# Developing a Mobile Patient-Reported Outcomes Version of the Common Terminology Criteria for Adverse Events Administration System to Capture Postradiation Toxicity in Oncology: Usability and Feasibility Study

**DOI:** 10.2196/27775

**Published:** 2022-04-12

**Authors:** Jody Underwood, Susan McCloskey, Ann Raldow, Amar Kishan, Chad Zalkin, Daniel Navarro, Lisa Scott Holt, Andrew Webb, Kathleen A Lynch, Thomas M Atkinson

**Affiliations:** 1 Intelligent Automation, Inc Rockville, MD United States; 2 Department of Radiation Oncology David Geffen School of Medicine at UCLA Los Angeles, CA United States; 3 Department of Psychiatry and Behavioral Sciences Memorial Sloan Kettering Cancer Center New York, NY United States

**Keywords:** neoplasms, patient outcome assessment, radiation oncology, toxicity, public health informatics, mobile apps, mobile health, mobile administration system, radiation therapy, eHealth

## Abstract

**Background:**

Accurate self-reported symptomatic toxicity documentation via the Patient-Reported Outcomes version of the Common Terminology Criteria for Adverse Events (PRO-CTCAE) is essential throughout cancer treatment to ensure safety and understand therapeutic efficacy. However, the capture of accurate toxicities from patients undergoing radiation therapy is challenging because this is generally provided only at the time of scheduled visits.

**Objective:**

This study seeks to establish the usability and feasibility of a mobile PRO-CTCAE Administration System (mPROS) to capture toxicities related to radiation therapy.

**Methods:**

English-speaking adult patients who were undergoing radiation therapy for cancer were enrolled and given a brief demonstration of the Say All Your Symptoms (SAYS) and Symptom Tracking Entry Program (STEP) interfaces of the mPROS app, followed by a patient-use phase where patient actions were observed as they navigated mPROS to enter toxicities. Patient feedback was captured via a semistructured interview and brief questionnaire.

**Results:**

We enrolled 25 patients (age: mean 60.7 years; females: n=13, 52%; White patients: n=13; 52%; non-Hispanic patients: n=19, 76%; college graduates: n=17, 68%). Patients almost equally preferred the SAYS (n=14, 56%) or STEP (n=11, 44%) interfaces, with 21 patients (84%) agreeing that they would use mPROS to report their symptoms to their health care team and 19 patients (76%) agreeing that they would recommend mPROS to others.

**Conclusions:**

The mPROS app is usable and feasible for facilitating the patient reporting of radiation therapy–related symptomatic toxicities. A revised version of mPROS that incorporates patient input and includes electronic health record integration is being developed and validated as part of a multicenter trial.

## Introduction

**Figure 1:**
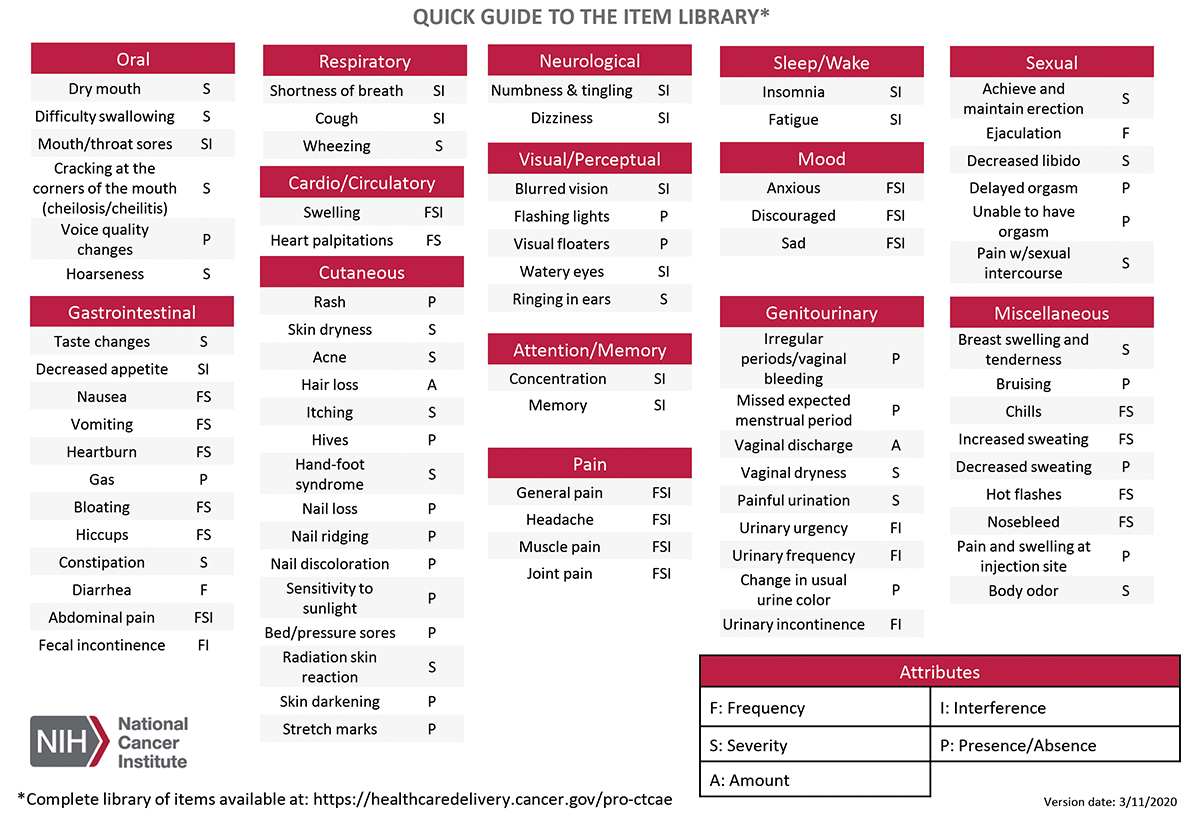
Patient-Reported Outcomes of the Common Terminology Criteria for Adverse Events item library version 1.0.

Toxicity reporting via the Common Terminology Criteria for Adverse Events (CTCAE) of the National Cancer Institute (NCI) is mandatory in oncology clinical trials to monitor patient safety and to understand the toxicity profiles of treatments [1]. Several studies have shown that symptomatic toxicities associated with anticancer treatments (eg, nausea and vomiting) are frequently underreported by health care providers, even when prospectively collected within clinical trials [2-5]. To address the issue of underestimating symptom toxicities related to cancer treatment, the NCI supported the creation of the Patient-Reported Outcomes (PRO) version of the CTCAE (PRO-CTCAE), referred to as PRO-CTCAE [6-10]. Version 1.0 of the PRO-CTCAE item library can be administered electronically [11] and includes 124 individual items representing the 78 toxicities, with multiple attributes captured for a given toxicity attribute question (eg, frequency, severity, and interference with usual or daily activities), as shown in Figure 1.

Although the PRO-CTCAE has preliminarily demonstrated promise in several areas [12-17], with the broad electronic delivery of the PRO-CTCAE showing clinical utility [18-21], this tool has not yet enjoyed widespread use in the radiation oncology setting [22-24]. The exposure of surrounding healthy tissue to the radiation field is largely unavoidable for patients with cancer who are treated with radiation therapy, making patient reports of symptomatic toxicities particularly important for this population. One barrier to using the PRO-CTCAE is that each radiation oncology clinic must develop infrastructure and support to administer a PRO-CTCAE assessment [25]. For example, administrative personnel in clinics creating custom PRO-CTCAE assessments using paper forms require additional time to enter the patient responses into digital systems. If the PRO-CTCAE is administered on a clinic-provided digital platform (eg, tablets), administrative support is required to answer questions from patients who do not routinely use technology (eg, for changing passwords) [26,27]. Although these complications are manageable, they may be reduced or eliminated through the development of mobile apps that do not require clinics to provide digital assessment platforms.

Categories of PRO-CTCAE items have not yet been established for commonly used cancer radiation treatments, necessitating that radiation oncologists select PRO-CTCAE items for a given treatment based on their clinical experience rather than a uniform standard. Additionally, the current practice is such that PRO-CTCAE assessments are not tailored to patient characteristics, previous assessment responses, or the area(s) of their body where they are receiving radiation therapy. Such patient-centered specificity in toxicity reporting would provide clinicians with crucial context that will assist in their interpretation of this patient-reported information. Further, for patients reporting PRO-CTCAE toxicities between visits or otherwise outside of the clinic setting, there is currently no means to remotely deliver relevant self-care information and suggestions (eg, strategies for treating mild skin symptoms). Patients may be more likely to engage in and complete the assessments if they know that they will learn something about their symptoms and how to treat them.

Despite the importance of leveraging the electronic capture of between-visit PRO information that can be incorporated directly into the electronic health record with respect to improving medical decision-making and prolonging overall patient survival [28-30], there are no cancer-specific mobile health apps available for this purpose [31,32]. Therefore, this study sought to complete a cross-sectional assessment of the usability and feasibility of the Mobile PRO-CTCAE Administration System (mPROS), an iOS- and Android-based smartphone app designed to address the aforementioned barriers through the tailoring of PRO-CTCAE assessments specifically to patients undergoing radiation therapy for cancer.

## Methods

### Recruitment

Patients with appointments in the radiation oncology clinics at Memorial Sloan Kettering Cancer Center (MSK), a tertiary NCI-designated Comprehensive Cancer Center in New York, NY, were screened by a clinical research coordinator (CRC) for initial eligibility. Patients were originally eligible for approach if they spoke English; were receiving radiation therapy in the head/neck, breast, or pelvic areas; and were aged 18 years or older. Delays related to patient accrual required the expansion of participant enrollment to those receiving radiation therapy for any disease type, despite mPROS only being equipped to address symptoms related to radiation in the head/neck, breast, and pelvic areas. The physicians attending to the patients were contacted for permission to approach patients who were determined to be eligible. Enrolled patients were given the option to end the study session at any time. Documented informed consent was not collected, as this study was deemed exempt by the Institutional Review Board at MSK.

### Mobile App

**Figure 2:**
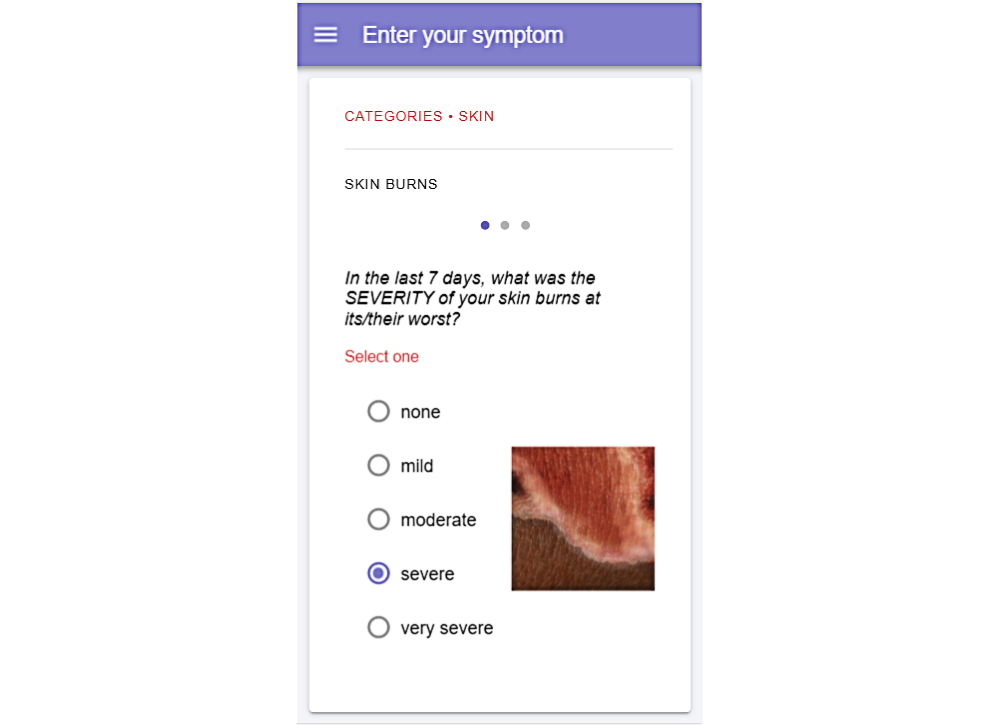
Symptom Tracking Entry Program interface.

**Figure 3:**
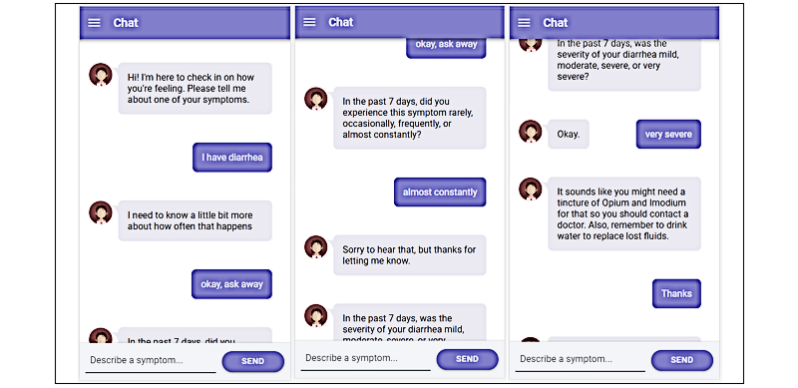
Chatbot on the Say All Your Symptoms interface.

The mPROS app uses 2 PRO-CTCAE assessment interfaces to collect data. The structured interface, called the Symptom Tracking Entry Program (STEP), delivers multimedia-supported PRO-CTCAE items relevant to the patient's radiation therapy (Figure 2). The second interface, Say All Your Symptoms (SAYS), leverages a virtual clinical assistant in the form of a chatbot to engage the patient in a text-based conversation to elicit patient responses to relevant PRO-CTCAE items in a comforting manner (Figure 3). Both interfaces are programmed to be responsive to symptoms related to radiation in the head/neck, breast, and pelvic areas for male and female patients.

### Procedure

The interactions during this research session took place in a quiet, private space in the MSK radiation oncology clinics and were audio-recorded to support the generation of a summary report. The CRC confirmed eligibility and determined if the patient was able to use a study-provided iOS- or Android-based smartphone on which the mPROS app was installed. Upon enrollment, the CRC collected demographic and treatment characteristics (eg, gender, age, education, race and ethnicity, the radiation treatment region, and the frequency of mobile phone app use) and provided an explanation about how mPROS works. Patients were then asked about their preferred operating system (ie, iOS or Android), the part of the body where they were currently being administered radiation, and 1 to 3 symptoms that they were currently experiencing or had recently experienced. This was followed by a brief (8-10 minutes) demonstration on how to use both interfaces of mPROS, with the CRC using symptoms not mentioned by the patient to avoid redundancy. Patients were given the opportunity to ask questions about mPROS and request a repeat demonstration of either mPROS interface.

Following the completion of the demonstration, patients were provided with the study smartphone based on their preferred operating system (ie, iOS or Android) and asked to use the mPROS app for approximately 5 to 10 minutes. The patients were asked to choose which interface they wanted to use first (ie, STEP or SAYS). The CRC guided the patients to progress through each interface but was instructed to provide additional assistance only when requested by the patients.

The patients’ explicit and implicit actions were observed and documented by the CRC (eg, the ability to switch between interfaces without assistance, issues related to progression through the app, app crashes, etc), and any aspects that appeared to be frustrating or time consuming were noted for the follow-up interview. All patient interactions with the mPROS app (ie, click location and time per page/interface in milliseconds) were captured by the mPROS system.

Upon completing the patient-use portion of the study within mPROS, the CRC conducted a semistructured interview in which patients were asked the following questions: (1) What features of mPROS did you like the most? (2) What features of mPROS did you like the least? (3) What suggestions do you have to improve mPROS? (4) Which mPROS interface did you prefer and why? (5) What symptoms or features of symptoms did you want to report in mPROS but could not? (6) How frequently do you think you would use the mPROS application? (7) What could we add to the mPROS application to make it more useful to you? (8) How would an application like mPROS help you in discussing your symptoms with your doctors? (9) Is there anything else that you would like to tell us about your experience with the mPROS application?

Patients were then asked to complete a brief 7-item questionnaire to indicate the degree to which they agreed with the following statements: (1) I would use the mPROS application to report symptoms to my health care team. (2) I would recommend mPROS to others. (3) I use other smartphone applications to track my symptoms. (4) I use other smartphone applications to track my daily activity and experiences. (5) I would be interested in connecting with other radiation patients through the mPROS application to share experiences. (6) I would like for the mPROS application to show me my symptom history over time. (7) I would like for the mPROS application to give me an option to use the phone camera to take a picture of my symptomatic area (eg, skin, mouth) to send to my doctor.

After the research interaction, patients were thanked for their participation, reimbursed US $50 for their time, and given the opportunity to spend additional time using the mPROS app if it was of interest to them. All audio recordings were destroyed within 48 hours of the research interaction.

## Results

### Patient Characteristics

**Figure 4:**
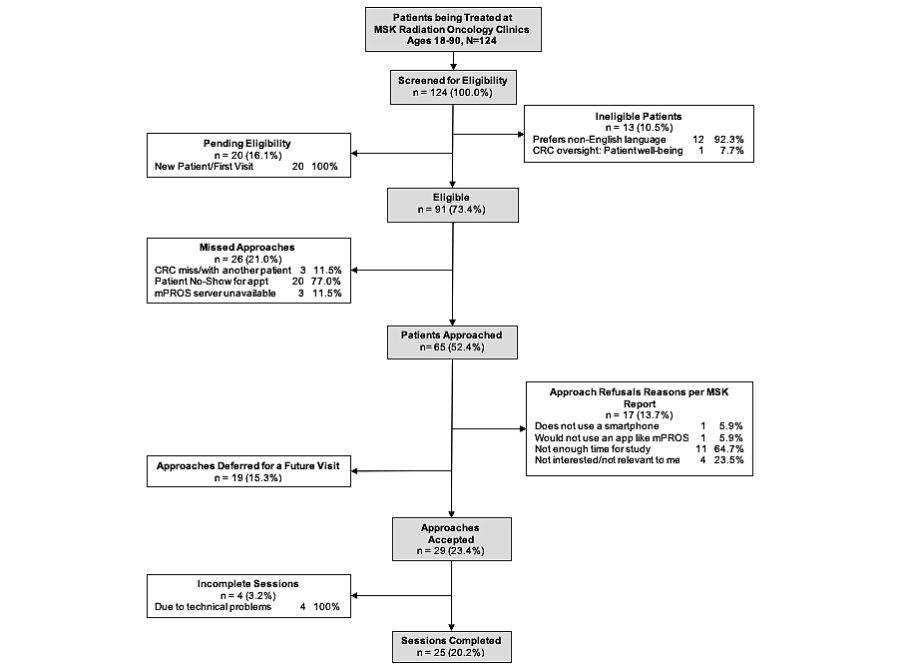
CONSORT (Consolidated Standards of Reporting Trials) diagram. appt: appointment; CRC: clinical research coordinator; MSK: Memorial Sloan Kettering Cancer Center; mPROS: Mobile Patient-Reported Outcomes.

A total of 124 patients were screened for eligibility between May and August 2019. Among them, 91 patients (73.4%) were found to be eligible; the CRC approached 65 (71.4%) of these 91 patients (Figure 4). The primary reason for not approaching patients was due to the rescheduling of appointments (n=20). Of the 65 approached patients, 29 (23.4%) accepted enrollment in the study, with a total of 25 patients (20.2%) completing the research interaction session. Further, 20 patients who were eligible did not show up for their scheduled appointment and thus could not be approached for participation, whereas 19 patients were approached and agreed to participate if they could complete the session during a future visit. The reasons for refusal included not enough time for the study (n=11, 64.7%), not interested/irrelevant (n=4, 23.5%), not using a smartphone (n=1, 5.9%), and not keen to use an app like mPROS (n=1, 5.9%).

The enrolled patients (age: mean 60.7 years; range 34-80 years) comprised 52% females and were mostly highly educated (68% college graduates or postgraduates), White (52%), and non-Hispanic (76%). The radiation regions included the brain (n=4), breast (n=4), chest/thorax (n=4), head/neck (n=4), lungs (n=2), lymph nodes (n=2), adrenal gland (n=1), liver (n=1), pelvic region (n=1), spine (n=1), and thighs (n=1), of which only 9 (ie, breast, head/neck, and pelvic region) were the targets for the current version of mPROS. All but 3 (12%) of the 25 patients indicated that they sometimes used smartphone apps, with 12 patients (48%) indicating that they “always” used smartphone apps. Furthermore, 20 patients (80%) currently use and prefer the iOS operating system, whereas 5 patients (20%) preferred using Android devices. The sample characteristics are included in Table 1.

**Table 1:** Demographic and clinical characteristics of the patients (N=25).

Characteristics		Value
**Age (years)**		
	Mean (SD)	60.7 (12.7)
	Median (range)	63.5 (34-80)
**Gender, n (%)**		
	Female	13 (52)
	Male	12 (48)
**Race, n (%)**		
	White	13 (52)
	Asian or Pacific Islander	3 (12)
	Black or African American	2 (8)
	Asian Indian	1 (4)
	Native American	1 (4)
	Mixed race	2 (8)
	Preferred not to answer	3 (12)
**Ethnicity, n (%)**		
	Non-Hispanic	19 (76)
	Hispanic	1 (4)
	Chose not to answer	5 (20)
**Education, n (%)**		
	High school graduate or less	5 (20)
	Post high school training/some college education	3 (12)
	College graduate/postgraduate	17 (68)
**Use of smartphone apps, n (%)**		
	Never	3 (12)
	Sometimes	4 (16)
	Often	6 (24)
	Always	12 (48)
**Cancer type, n (%)**		
	Breast^a^	4 (16)
	Head/neck^a^	4 (16)
	Chest/thorax	4 (16)
	Brain	4 (16)
	Lungs	2 (8)
	Underarm lymph nodes	2 (8)
	Pelvic region^a^ (male)	1 (4)
	Spine	1 (4)
	Thighs	1 (4)
	Liver	1 (4)
	Adrenal gland	1 (4)
	Pelvic region^a^ (female)	0 (0)

^a^Cancer type specifically targeted in the current mPROS (Mobile Patient-Reported Outcomes version of the Common Terminology Criteria for Adverse Events Administration System) app.

### Demonstration Phase

Patients generally understood the use of mPROS during the demonstration phase; no patients requested a repeat demonstration of the mPROS features. However, 3 patients had questions regarding treatment management suggestions (eg, 2 types of ibuprofen suggested for pain rather than 1 type), and 1 patient asked whether text had to be entered into the final open-ended text box for gastrointestinal symptoms for the symptom form to be “complete.”

### Patient-Use Phase

The mean time to complete the patient-use phase was 19 minutes and 33 seconds (range 7 minutes and 22 seconds to 39 minutes and 17 seconds). All but 2 of the participants were able to navigate between STEP and SAYS without assistance from the CRC.

### Semistructured Interviews

Table 2 includes a summary of patient preferences, perceived mPROS benefits, suggestions for mPROS improvement, and general feedback on the app. The majority of the patients indicated they would use mPROS on a regular basis (n=12) or when they noticed a marked change in their symptoms (n=13). Patient statements included “I would use it every day because of my chronic symptoms.”

When asked about their suggestions to improve mPROS, 8 patients suggested the addition of a feature to send mPROS communications directly to their doctor, preferring a 2-way confirmation of data sent and received. The addition of a feature for patients to enter lifestyle-related information (eg, current medications, treatments, diet, mental health, and exercise routines) was suggested by 7 patients. Another suggestion given by 4 patients was to have an alert system in place for any instance of a symptom being reported as “very severe,” and 3 patients spontaneously suggested adding a feature to upload photographs of their skin. The inclusion of a spell-check feature in the SAYS interface was suggested by 3 patients. Of these 3 patients, 2 suggested having SAYS confirm a symptom entry before advancing to the related questions or having it suggest a symptom if the user’s input was not recognized (eg, “you said ‘headache’ – did you mean head pain?”). Two patients suggested including legal language before reporting symptoms (eg, “If you are experiencing an emergency, please call 911 or contact your doctor”). One patient suggested a “sent confirmation/read receipt” feature be added to the app, and another patient suggested the addition of an option to enter medications or treatments already taken for a given symptom.

With respect to using mPROS to help with discussing symptoms with their doctor, 14 patients mentioned the advantage of being able to record and recall symptoms over time, with 9 patients indicating that mPROS would help them to report symptoms over time, 7 mentioning that using mPROS would help their doctor or treatment team, and 4 stating that they are currently recording and maintaining their own physical set of symptom notes that would otherwise be documented in the mPROS app.

The mPROS app provided artistically rendered images to help patients understand the skin burn severity levels (mild, moderate, severe, and very severe). When asked about the skin severity images, 17 of the 25 patients (68%) found these to be helpful, and 18 patients indicated that the binary (ie, light versus dark) skin tone was insufficient, with 2 patients spontaneously raising this point prior to the interview question. The inclusion of additional context was requested by 3 patients when selecting skin color during patient setup, as the images do not appear until later, and they appear only if skin symptoms are included in the STEP interface. Another patient suggested asking the skin color question only when specifically indicating that a patient is experiencing a skin-related symptom. Additionally, 6 patients did not find the skin images to be helpful, citing that the skin burn images were not relevant to them either because the choices within skin effects did not include burns or because the reference images did not represent their healthy skin color. These patient responses included “I didn’t find these to be helpful – I have discoloration, not burns. It would be better to upload pictures of your skin as you go through radiation” and “No – they don’t look like skin to me. Light and dark skin was not specific enough. You need more gradations or have us pick from a continuum.”

**Table 2:** Patient preferences, perceived benefits, general feedback, and suggestions for improvement identified from the semistructured interviews (N=25).

Survey items			Value, n (%)
**Interface choice**			
	Used SAYS^a^ first	13 (52)	
	Used STEP^b^ first	12 (48)	
**Interface preference**			
	SAYS	14 (56)	
	STEP	11 (44)	
**Interest in future use**			
	Would use mPROS^c^ on ad hoc basis depending on symptom changes	13 (52)	
	Would use mPROS on regular basis	12 (48)	
**Perceived benefits of mPROS**			
	Ability to record and recall symptoms over time	14 (56)	
	Assistance with recording symptoms over time	9 (36)	
	Will assist treatment team	7 (28)	
**Most liked feature**			
	SAYS chatbot	9 (36)	
	Specifying body areas	6 (24)	
	Speed/convenience of completing symptom reporting using the app versus completing it in the doctor's office	5 (20)	
	Ability to choose between methods of reporting symptoms	3 (12)	
	No response	2 (8)	
**Least liked feature**			
	mPROS not recognizing symptom	7 (28)	
	Confusion over when to swipe/tap “next” to enter more attributes	6 (24)	
	Texting	4 (16)	
	No response	8 (32)	
**Patient suggestions to make mPROS more useful**			
	Ability to send reports directly to clinicians	8 (32)	
	Ability to enter medications, treatments, diet, mental health, and exercise	7 (28)	
	Alert system for very severe symptoms	4 (16)	
	Less verbose questions/chat language	4 (16)	
	Spell-check for SAYS chatbot	3 (12)	
	Ability to upload skin photos	3 (12)	
	SAYS confirmation of symptom entry	2 (8)	
	Inclusion of legal language before reporting symptoms	2 (8)	
	Send confirmation/read receipt to clinicians	1 (4)	
	Specifying skin tone for skin-related symptoms	1 (4)	
	Use of a 1-5 scale rather than asking patients to type verbal descriptions	1 (4)	
	Additional safety features for logging into the app	1 (4)	
**Severity image feedback**			
	Binary skin tone (ie, light versus dark) insufficient	18 (72)	
	Images generally helpful	17 (68)	
	Not relevant due to lack of burns or not representing healthy skin color	6 (24)	
**General feedback and issues**			
	Confused about right-swipe navigation of attribute questions	18 (72)	
	Helpful completion icons	9 (36)	
	Attempted to enter symptoms not associated with mPROS radiation sites	7 (28)	
	Struggled with text entry	6 (24)	
	Body areas too large to show where symptoms occurred	3 (12)	
	Problems finding home screen	2 (8)	

^a^SAYS: Say All Your Symptoms.

^b^STEP: Symptom Tracking Entry Program.

^c^mPROS: Mobile Patient-Reported Outcomes version of the Common Terminology Criteria for Adverse Events Administration System.

### Patient mPROS Questionnaire

Among the 25 patients, 21 (84%) agreed or strongly agreed that they would use the mPROS app to report their symptoms to their health care team, and 19 (76%) agreed or strongly agreed that they would recommend mPROS to others. Approximately half the patients do not currently use other mobile apps to track their symptoms (n=12, 48%), with the majority indicating they do not use other mobile apps to track other activities (n=16, 64%). When asked whether they would be interested in connecting with other radiation patients through mPROS, only 10 (40%) patients were interested in such a feature. Except for 1 patient, the remaining 24 (95%) would like for mPROS to show symptom history over time and provide an option to send a photograph of their symptomatic area using the phone camera.

## Discussion

### Principal Findings

The underreporting of symptomatic toxicities throughout the course of radiation treatment can lead to the underestimation of the absolute rate of toxicity, which can immediately impact clinical decision-making. More accurate patient-tailored reporting of toxicities can lead to improved personalized care and quality of life for patients with cancer having difficulty with any treatment. This study sought to establish the usability and feasibility of mPROS, a mobile app that was designed to measure symptomatic toxicities via PRO-CTCAE and was specific to patients undergoing radiation therapy, in a sample of patients from radiation oncology clinics at a tertiary cancer center. The mPROS app was generally liked, with the majority of patients indicating they would use the app to report symptoms to their health care team. Some patients indicated that they liked the speed or convenience of reporting symptoms on their phone versus doing it in the doctor’s office. Others liked having a choice of how to report symptoms.

Using mPROS to capture patient-reported toxicities is feasible; only 17 of the original 91 (18.7%) patients who were determined eligible for the study refused to participate (Figure 1). Of the 25 patients who participated, 21 (84%) indicated that they would use this app to report symptoms to their health care team and 19 (76%) stated that they would recommend mPROS to others. Limited training (ie, 8-10 minutes) is required for orienting patients to use mPROS; no patients requested additional demonstrations of the mPROS app prior to initiating the patient-use phase. Despite our patients’ median age of 63.5 years and the inclusion of 11 patients aged 65 years or older (range 34-80 years), we observed no age-related challenges in the use of mPROS. This is consistent with recent work that established the feasibility of electronic geriatric health assessment in patients aged 65 years or older in a multi-institutional setting [33].

Using a qualitative methodology to establish the usability of mPROS is a strength of our study [34]. These patient-centered interactions provided important feedback for the refinement of mPROS and demonstrated that there is a need to have a personal app for reporting symptoms; moreover, the SAYS chatbot is desired by patients who are comfortable with texting, and there are symptoms that are not caused directly by radiation that patients still experience and want to report, such as headaches and nausea. Additionally, the majority of patients indicated that the skin severity images were insufficient and did not represent their healthy skin color. Suggestions were made to replace the binary (ie, light versus dark) skin tones with a sliding skin tone scale. The next version of mPROS will contain other recommended features that will make it more useful to the patients.

There are a number of limitations to this study. This was a single-site study completed in a tertiary cancer center with a mostly highly educated study sample with limited Hispanic representation. Additionally, delays related to patient accrual necessitated the expansion of eligibility criteria to any English-speaking patient aged 18 years or older who was receiving radiation therapy for any disease type. The version of mPROS that was used for testing only included body map selections and symptoms related to receiving radiation in the breast, head/neck, or pelvic region. Enrolled patients who were receiving radiation in a different region (n=16) were asked to select a region that was proximal to their radiation site, and several of their suggestions involved expanding mPROS to include their specific radiation sites (n=4). Despite this version of mPROS not including their radiation sites, all but 1 (n=15, 94%) of these participants indicated that they would use this app to report symptoms to their health care team. Finally, as mPROS was evaluated on a study-provided smartphone during a single session with each participant, we did not consider it appropriate to assess the acceptability of the app at this time; acceptability will be analyzed as part of an ongoing multicenter clinical trial.

### Conclusions

The mPROS app is a usable and feasible tailored assessment for patients to report symptomatic toxicities related to their radiation therapy. Using patient input from this study, a revised version of mPROS that includes electronic health record integration is being developed and validated as part of a multicenter clinical trial (National Institutes of Health/NCI Small Business Innovation Research Phase 2 Contract #75N91020C00027). The seamless electronic documentation of these patient-reported symptomatic toxicities will ensure that this information is considered as part of the clinical decision-making process, and this may ultimately improve patient outcomes.

## Abbreviations

CRC: clinical research coordinator
CTCAE: Common Terminology Criteria for Adverse Events
mPROS: Mobile Patient-Reported Outcomes version of the Common Terminology Criteria for Adverse Events Administration System
MSK: Memorial Sloan Kettering Cancer Center
NCI: National Cancer Institute
PRO: Patient-Reported Outcomes
PRO-CTCAE: Patient-Reported Outcomes version of the Common Terminology Criteria for Adverse Events
SAYS: Say All Your Symptoms
STEP: Symptom Tracking Entry Program
